# Transcriptome analysis and functional validation reveal that VvMYB1 is a positive regulator of drought tolerance in grape

**DOI:** 10.3389/fpls.2026.1853060

**Published:** 2026-06-15

**Authors:** Ziyi Wu, Mengran Duan, Guangshuo Li, Ying Zhao

**Affiliations:** 1 School of Enology and Horticulture, Ningxia University, Yinchuan, China; 2 Engineering Research Center of Grape and Wine, Ministry of Education, Ningxia University, Yinchuan, China; 3 Anhui Province Key Laboratory of Crop Integrated Pest Management, School of Plant Protection, Anhui Agricultural University, Hefei, China

**Keywords:** drought stress, physiological indicators, stress resistance genes, transcriptome analysis, VvMYB1

## Abstract

**Introduction:**

Drought stress severely constrains grape growth and productivity, yet the molecular mechanisms underlying drought adaptation remain incompletely understood. This study aimed to identify key transcriptional regulators involved in grape drought responses and to evaluate their functional roles.

**Methods:**

To address this, callus tissues derived from *Vitis vinifera* were subjected to PEG-simulated drought stress, and samples were collected at 0, 5, and 10 days for transcriptome sequencing. Differential expression analysis was performed to identify drought-responsive genes and transcription factor families. Based on expression patterns, VvMYB1 was selected for further functional characterization. Transgenic callus overexpressing *VvMYB1* was generated to assess its role under drought stress.

**Results:**

Transcriptome analysis identified 48 transcription factor families involved in drought response, among which the MYB family was the most abundant. Functional analyses showed that OE-*VvMYB1* callus exhibited enhanced drought tolerance compared with WT, as evidenced by significantly increased activities of SOD, POD, and CAT, as well as higher Pro accumulation. In contrast, MDA and H_2_O_2_ levels were reduced. Moreover, the expression levels of several stress-responsive genes were significantly upregulated in OE-*VvMYB1* lines.

**Discussion:**

These results indicate that VvMYB1 functions as a positive regulator in grape drought response. This study provides new insights into the transcriptional regulation of drought tolerance in grape and identifies VvMYB1 as a promising candidate gene for improving stress resilience.

## Introduction

Grape (*Vitis vinifera* L.) is one of the most widely cultivated fruit crops worldwide, with a well-established industrial chain encompassing fresh consumption, winemaking, and deep processing. It plays a crucial role in global agriculture and the food industry (Jaillon et al., 2007; Gambetta et al., 2020). In arid and semi-arid regions such as Ningxia in northwestern China, limited precipitation, high evaporation rates, and scarce water resources make drought a major environmental constraint on agricultural production (Su et al., 2015). As a key regional crop, grape growth and yield are highly sensitive to water availability. Prolonged or intermittent drought stress can significantly inhibit shoot growth and leaf expansion, reduce photosynthetic efficiency, and impair carbon assimilation and biomass accumulation (Fraga et al., 2016). Moreover, water deficit disrupts fruit development and quality formation, leading to reduced berry size and yield, as well as alterations in sugar-acid balance and phenolic composition, ultimately affecting both the market value and flavor quality of table and wine grapes (Gambetta et al., 2020; Yu et al., 2023). Therefore, elucidating the molecular regulatory networks underlying grape responses to drought stress and identifying key regulatory factors are essential for developing drought-tolerant cultivars and promoting the sustainable development of the grape industry.

To date, numerous MYB transcription factors (TFs) have been identified as key regulators of drought tolerance in various plant species (Baldoni et al., 2015). In *Arabidopsis thaliana*, AtMYB60 promotes root elongation to enhance water uptake during early drought stress, while its function is closely associated with abscisic acid (ABA)-mediated stomatal regulation (Oh et al., 2011). In tomato (*Solanum lycopersicum*), the SlMYB55 regulates drought and salt stress responses by modulating ABA biosynthesis and signaling, thereby influencing the expression of genes associated with stress tolerance, flowering time, and floral development (Chen et al., 2022). Similarly, the SlMYB1L enhances drought tolerance via an ABA-dependent pathway (Liu et al., 2025). Overexpression of *PlMYB108* from *Paeonia lactiflora* in tobacco significantly increases flavonoid accumulation, antioxidant enzyme activities, and photosynthetic capacity, indicating its role in drought stress responses (Wu et al., 2021). Likewise, PtoMYB142 in *Populus tomentosa* improves drought tolerance by regulating cuticular wax biosynthesis (Song et al., 2022). In rice (*Oryza sativa*), overexpression of *OsMYB6* enhances tolerance to both drought and salt stresses (Tang et al., 2019; Qu et al., 2022). Furthermore, GmMYB14 improves drought tolerance and yield performance in soybean (*Glycine max*), and GhMYB36 enhances resistance to drought and apical blight in cotton (*Gossypium hirsutum*), whereas its silencing leads to increased sensitivity (Chen et al., 2021; Liu et al., 2022a).

BnaMYB52 acts as a negative regulator of drought tolerance in *Brassica napus*, modulating the expression of downstream target genes *BnaMYB96* and *BnaMYB30*, and thereby negatively influencing drought resistance by regulating water loss through stomatal behavior and cuticular permeability (Wu et al., 2026). Drought stress activates a phosphorylation-dependent signaling cascade in which ZmCRK5A phosphorylates the MYB repressor ZmSMH4 to relieve its inhibition of *ZmKCH1* expression, thereby modulating stomatal dynamics and enhancing drought tolerance while maintaining grain yield in maize (Ma et al., 2026). PdbMYB44 enhances drought tolerance in Shanxin poplar (*Populus davidiana × P. bolleana*) by directly repressing PP2C transcription, thereby activating the SnRK2.6-SLAC1 cascade to drive ABA-dependent stomatal closure and improve water retention and stress adaptation (Yang et al., 2026). Drought-induced CsMYB44/73 repress *CsmiR408* by binding to MBS elements in its promoter, thereby relieving inhibition of the target gene *CsLAC13* to promote lignin accumulation while suppressing flavonoid biosynthesis, ultimately enhancing drought tolerance in tea plants (Jia et al., 2026). TaMYB96-2D enhances drought tolerance in wheat (*Triticum aestivum*) by directly activating diketone biosynthetic genes through binding to CAACCA motifs, thereby promoting cuticular wax accumulation and the glaucous phenotype, which reduces water loss and improves stress resilience (Li et al., 2026).

In addition to these regulatory roles in drought adaptation, MYB have also been widely implicated in controlling phenylpropanoid metabolism, which contributes to stress tolerance through antioxidant activity. NtMYB308 functions as a repressor of phenylpropanoid metabolism in tobacco by directly suppressing anthocyanin and lignin biosynthetic genes, thereby reducing antioxidant capacity and cell wall reinforcement and ultimately compromising resistance to fungal pathogens (Singh et al., 2026). McMYB52 functions as a key positive regulator of purple tepal formation in *Michelia crassipes* by directly activating *McCCoAOMT* and orchestrating phenylpropanoid metabolism, thereby coupling flavonoid and lignin biosynthesis with epidermal cell morphogenesis (Yu et al., 2026). GmMYB84 confers cadmium tolerance by activating phenylpropanoid-related pathways and antioxidant defenses, thereby mitigating oxidative stress and sustaining *Glycine max* growth under Cd exposure (Gong et al., 2026).

In grape, MYB have also been implicated in drought stress responses. VyMYB24, isolated from the drought-tolerant wild species *Vitis yanshanesis*, positively regulates drought tolerance and plant development (Zhu et al., 2022). Similarly, VhMYB2 and VhMYB15 from the hybrid rootstock ‘Beta’ (*Vitis labrusca × Vitis riparia*) enhance drought and salt tolerance when overexpressed in *Arabidopsis* (Ren et al., 2023b, Han et al., 2024). In addition, VaMYB14 from cold-tolerant *Vitis amurensis* improves drought and cold tolerance by activating lipid transfer protein genes and enhancing reactive oxygen species scavenging (Fang et al., 2024). However, most of these studies have focused on MYB genes from wild grape species or hybrid rootstocks, while the roles of MYB transcription factors in drought responses of cultivated grape (*Vitis vinifera*) remain relatively less explored. In particular, the function of *VvMYB1* in drought stress response has not yet been characterized.

To address this gap, transcriptome sequencing was conducted on callus tissues of ‘Cabernet Sauvignon’ (*Vitis vinifera*) subjected to Polyethylene glycol (PEG)-induced stress at multiple time points. Comparative analysis revealed that transcription factor families were prominently enriched among the differentially expressed genes, with MYB members showing the most pronounced response patterns. Among them, *VvMYB1* was significantly and consistently induced under drought conditions, and was therefore selected for further functional characterization.

We hypothesize that *VvMYB1* functions as a key regulator of drought tolerance in grape by modulating stress-responsive gene expression. To test this hypothesis, transgenic callus overexpressing *VvMYB1* was generated and evaluated under drought stress. By integrating transcriptomic profiling with functional validation, this study aims to elucidate the regulatory mechanisms underlying drought responses in grape.

## Materials and methods

### Plant materials

Callus tissues derived from ‘Cabernet Sauvignon’ (*Vitis vinifera*) were used as the experimental material in this study. The callus cultures have been maintained in our laboratory through continuous subculture. The cultures were grown under controlled conditions at 25 °C with a 16 h light/8 h dark photoperiod and a light intensity of approximately 90 μmol·m^-2^·s^-1^.

### Screening of PEG concentration for drought treatment

PEG is widely used to simulate drought stress in studies of plant stress responses. Based on previous studies, five PEG concentration gradients (0%, 5%, 10%, 15%, and 20%) were established to mimic drought conditions. Callus tissues were transferred onto media containing different PEG concentrations and cultured for 0–10 days. Growth status of the callus was monitored to determine the appropriate PEG concentration for subsequent experiments.

### Transcriptome sequencing

Based on the results of PEG concentration screening, a B5 medium supplemented with PEG was used as the drought treatment medium. WT callus tissues were cultured under drought conditions for 0, 5, and 10 days, with three biological replicates for each time point. After treatment, samples was frozen in liquid nitrogen and ground to a fine powder and total RNA was extracted using Trizol. NEBNext Ultra II RNA Library Prep Kit (New England Biolabs, Inc, USA) was used to construct cDNA library. The sample was sequencing on the Illumina NovaSeq platform by Shanghai Majorbio Co., Ltd, using a paired-end 2 × 150 bp strategy. The Q30 rate exceeded 95%, and more than 7.1 Gb of clean data were generated for each sample.

### Transcriptome data processing

Raw sequencing data were subjected to quality control to remove low-quality reads using Trimmomatic (Bolger et al., 2014). Clean reads were then aligned to the reference genome of *Vitis vinifera* (GCF_030704535.1) by HISAT2 for downstream analysis (Kim et al., 2019).

### Gene expression analysis and identification of differentially expressed genes

Gene expression levels were calculated as fragments per kilobase of exon per million mapped reads (FPKM) using Cufflinks (v2.1.1) (Trapnell et al., 2010). Correlation analysis among samples was performed using R software. Differentially expressed genes (DEGs) were identified using edgeR (Robinson et al., 2010). Genes with log_2_(fold change) ≥ 1 and adjusted *P*-value < 0.05 were considered significantly differentially expressed. Heatmaps and Venn diagrams were generated using TBtools-II (Chen et al., 2023).

### GO and KEGG enrichment analysis

Functional enrichment analysis of DEGs was conducted using Gene Ontology (GO) and Kyoto Encyclopedia of Genes and Genomes (KEGG) databases. GO enrichment analysis was performed using GOATOOLS, and KEGG pathway enrichment analysis was conducted using R scripts clusterProfiler (Yu et al., 2012; Klopfenstein et al., 2018). Terms with an adjusted *P*-value < 0.05 were considered significantly enriched.

### Transcription factor analysis

Transcription factors were identified based on conserved domain information in gene products. The Plant Transcription Factor Database (PlantTFDB) was used for TF annotation, combined with BLAST analyses with an e-value threshold of 1e-5. Identified TFs were classified into different families, and MYB transcription factors were further analyzed for their expression patterns to evaluate their potential involvement in drought response.

### Construction of over-expressing *VvMYB1* transgenic callus

Total RNA was extracted from WT callus tissues using an RNA extraction kit and reverse-transcribed into cDNA. The CDS of *VvMYB1* was obtained from the NCBI, and gene-specific primers were designed (Table. S1). The CDS was amplified using cDNA as a template, and the PCR products were verified by agarose gel electrophoresis and sequencing. The confirmed PCR fragments were then cloned into the pCXSN vector using homologous recombination to generate the overexpression construct (pCXSN-*VvMYB1*), which was subsequently transformed into *Escherichia coli* competent cells. Positive clones were selected and verified, and the recombinant plasmid was extracted and introduced into *Agrobacterium tumefaciens* strain GV3101.

*Agrobacterium tumefaciens* carrying the recombinant plasmid was cultured to an OD_600_ of 0.6-0.8 and resuspended in B5 liquid medium supplemented with 100 μM acetosyringone (AS). Grape callus tissues were immersed in the bacterial suspension for 10 min with gentle agitation to ensure uniform infection. After infection, the calli were blotted dry on sterile filter paper and co-cultivated on B5 medium supplemented with 100 μM AS in the dark at 26 °C for 36 h. Following co-cultivation, the calli were washed three times with sterile water containing 300 mg/L cefotaxime to remove residual *Agrobacterium*, and then transferred to B5 medium supplemented with 300 mg/L cefotaxime and 300 mg/L carbenicillin for 5 days in the dark at 26 °C. Subsequently, the calli were transferred to selection medium (B5) containing 10 mg/L hygromycin (Hyg) and cultured in the dark until resistant calli were obtained.

### Identification of OE-*VvMYB1* transgenic callus

Transgenic callus OE-*VvMYB1* was selected and maintained on B5 medium supplemented with Hyg (10 mg/L), based on the Hyg resistance gene present in the pCXSN vector. To confirm the presence of the transgene, total RNA was extracted from putative transgenic callus and reverse-transcribed into cDNA. PCR amplification of the *Hyg* gene was performed, and the products were analyzed by 1% agarose gel electrophoresis. The presence of a specific amplification band indicated successful transformation. Furthermore, quantitative real-time PCR (qRT-PCR) was conducted to evaluate the expression level of *VvMYB1* in transgenic callus. Three biological replicates and two technical replicates were included for each sample. Relative gene expression levels were calculated using the 2^−ΔΔCt method.

### Drought treatment and phenotypic analysis of OE-*VvMYB1* transgenic callus

WT and OE-*VvMYB1* callus tissues were subjected to drought treatment for 0 and 10 days, with WT serving as the control. Morphological changes were recorded by photographing the callus before and after treatment. Following drought treatment, physiological and biochemical parameters were measured using commercial assay kits, including the activities of catalase (CAT, Solarbio, BC0205), superoxide dismutase (SOD, Solarbio, BC5165), and peroxidase (POD, Solarbio, BC0095), as well as the contents of proline (Pro, Solarbio, BC0295), hydrogen peroxide (H_2_O_2_, Solarbio, BC3595), and malondialdehyde (MDA, Solarbio, BC0025). The results were statistically analyzed to compare stress responses between WT and OE-*VvMYB1* callus. In addition, total RNA was extracted from treated callus tissues and reverse-transcribed into cDNA. Quantitative reverse transcription PCR (qRT-PCR) was performed to analyze the expression levels of drought-responsive genes, including *VvERD14*, *VvDREB2A*, *VvNAC72*, *VvSRK2A*, and *VvCBL1*. we used *VvActin* as the reference gene for normalization. Primer specificity was confirmed by melting curve analysis, and only primers showing single-peak melting curves were used for qRT-PCR analysis. Relative gene expression levels were calculated using the 2^ΔΔCt method. Gene-specific primers are listed in **Supplementary Table 1**.

### Statistical analysis

All experiments were performed with three biological replicates, and the results are presented as mean ± standard deviation (SD). Statistical analyses were conducted using SPSS 20.0 software. Differences between two groups were evaluated using Student’s *t*-test, while comparisons among three or more groups were performed using one-way analysis of variance (ANOVA) followed by Duncan’s test. Differences were considered statistically significant at *P* < 0.05.

## Results

### Screening of PEG concentration for drought treatment

**Figure 1:**
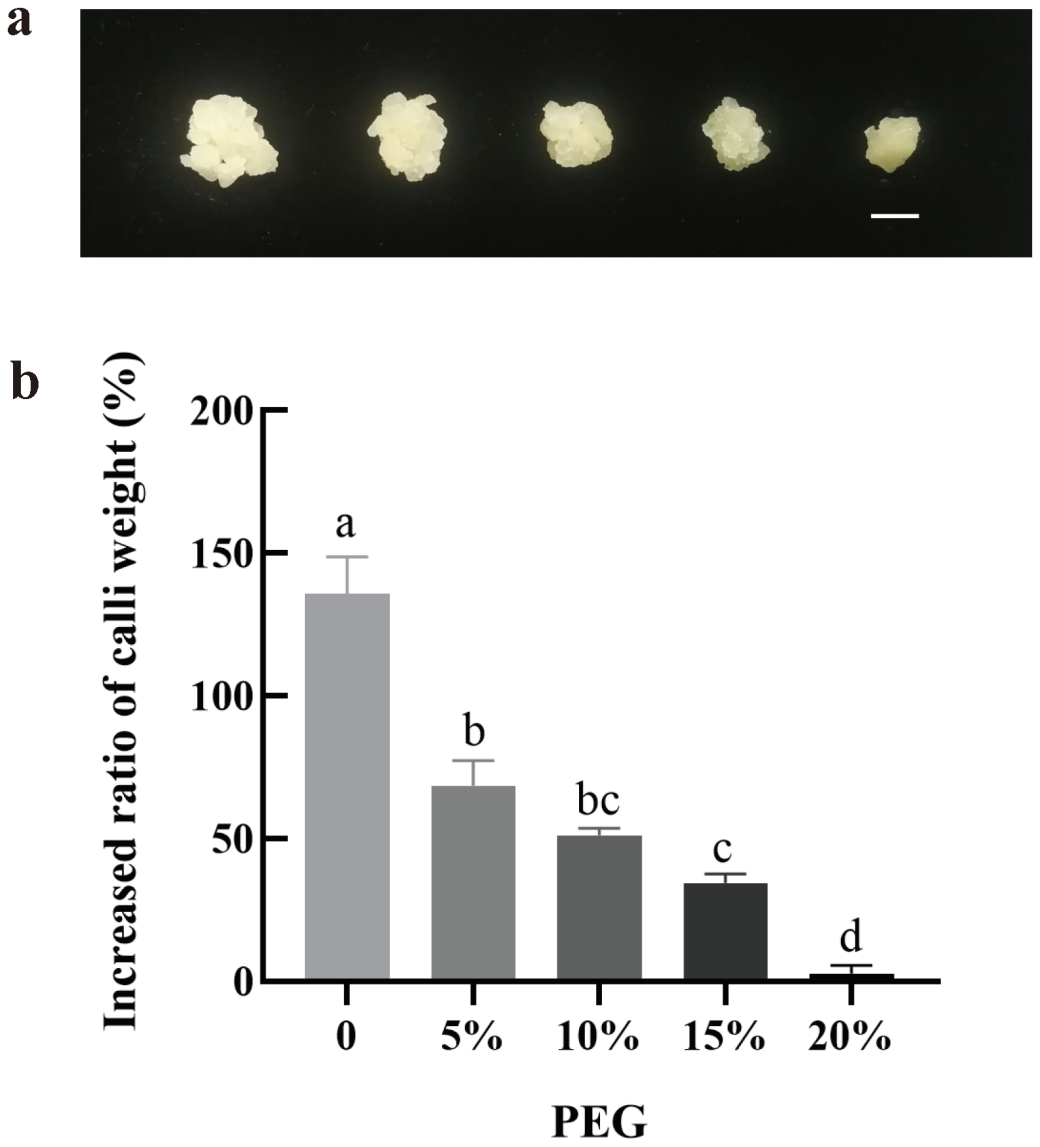
Effects of different concentrations of PEG treatment on grape callus growth. **(a)** From left to right, the PEG concentrations are 0%, 5%, 10%, 15%, and 20%. Scale: 1.0 cm. **(b)** Percentage increase in callus weight after PEG treatment at different concentrations. Weight increase percentage was calculated as (Wt − W_0_)/W_0_ × 100%, where W_0_ is the initial weight and Wt is the weight after culture. Data represent the mean ± SD of three biological replicates. Different letters indicate significant differences (*P* < 0.05).

Under different PEG treatment concentrations, the growth of grape callus was progressively inhibited with increasing PEG levels (**Figure 1**). At 5% PEG, callus growth was reduced to approximately 50% of that under normal conditions, indicating a significant inhibitory effect. When the PEG concentration reached 20%, callus growth was almost completely suppressed, with little to no growth observed. Based on these results, 5% PEG was selected for subsequent experiments, as it effectively inhibited callus growth while maintaining cell viability and physiological integrity.

### Correlation analysis of gene expression patterns

**Figure 2:**
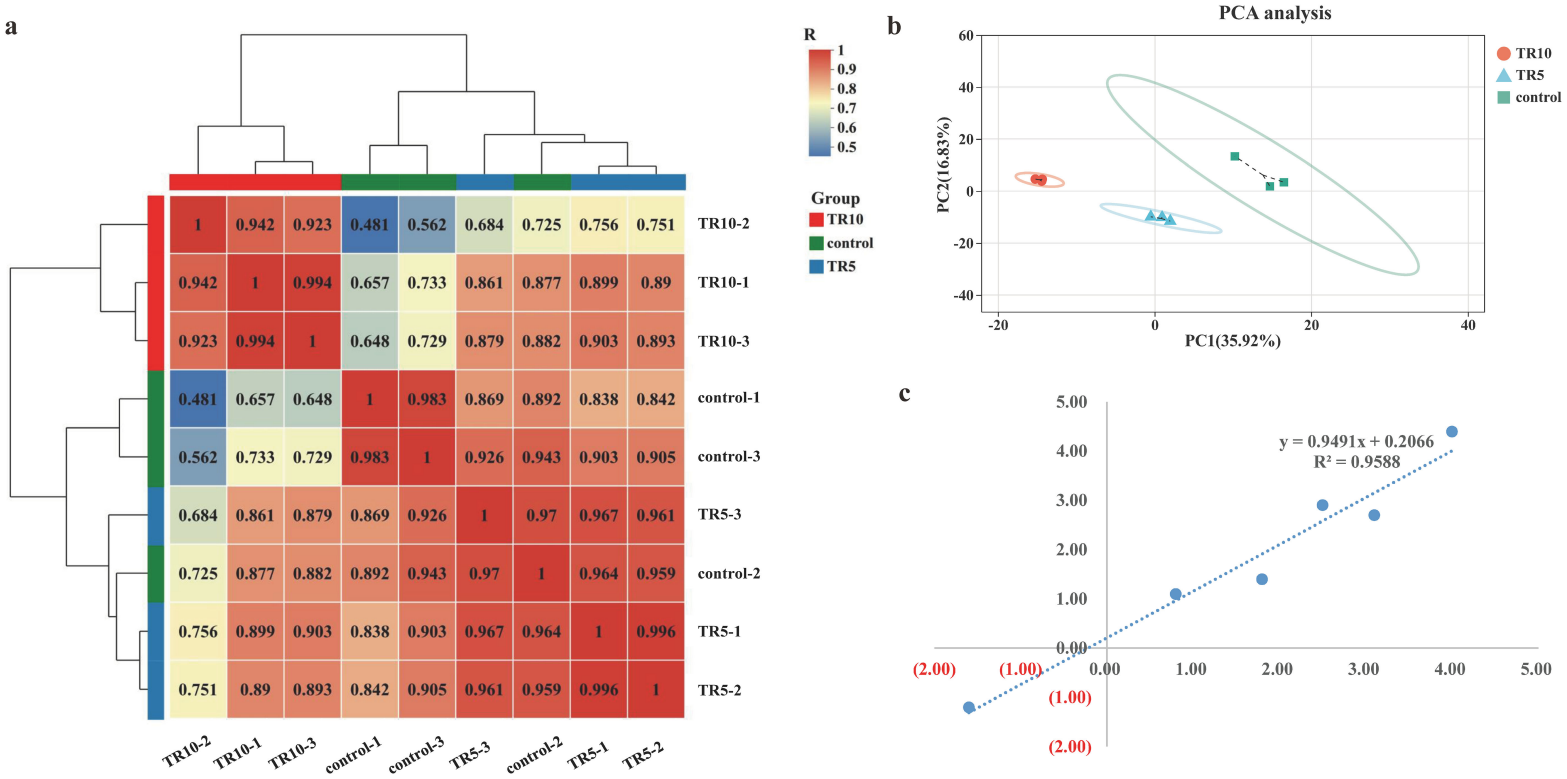
Correlation analysis between samples and principal component analysis. **(a)** Pearson correlation coefficients heatmap. The sample names are shown on the right and bottom sides of the heatmap, while the dendrograms on the left and top represent the hierarchical clustering of samples. Different colored squares indicate the degree of correlation between two samples. The control group represents normally watered plants, whereas TR5 and TR10 indicate drought treatment for 5 and 10 days, respectively. Each treatment contained three biological replicates. **(b)** Principal component analysis (PCA) of RNA-seq samples. Each point represents an individual sample, and the distance between points reflects the similarity among samples, with shorter distances indicating higher similarity. PC1 and PC2 represent the first and second principal components, respectively, and the percentages indicate the proportion of total variance explained by each component. **(c)** Validation of RNA-seq data by qRT-PCR. The expression patterns obtained by qRT-PCR were consistent with the RNA-seq data, showing a strong positive correlation.

To assess the reliability and reproducibility of the transcriptome data, Pearson correlation coefficients were calculated among all samples based on gene expression levels (FPKM values). In this study, the correlation coefficients among the three biological replicates within each group were all higher than 0.892 (**Figure 2A**), demonstrating good consistency and reliability of the data for subsequent analyses. Principal component analysis (PCA) was further performed on the nine samples to evaluate the overall variation and relationships among different treatment groups. As shown in **Figure 2B**, biological replicates within each group clustered closely together, whereas samples from different groups were clearly separated. Six DEGs were selected for qRT-PCR validation. The qRT-PCR results showed expression patterns consistent with the RNA-seq data, with a strong positive correlation (R² = 0.95), indicating the reliability of the transcriptome sequencing results (**Figure 2C**). These results indicate distinct transcriptional differences between treatments and confirm the robustness of the experimental design.

### Identification of differentially expressed genes under drought stress

**Figure 3:**
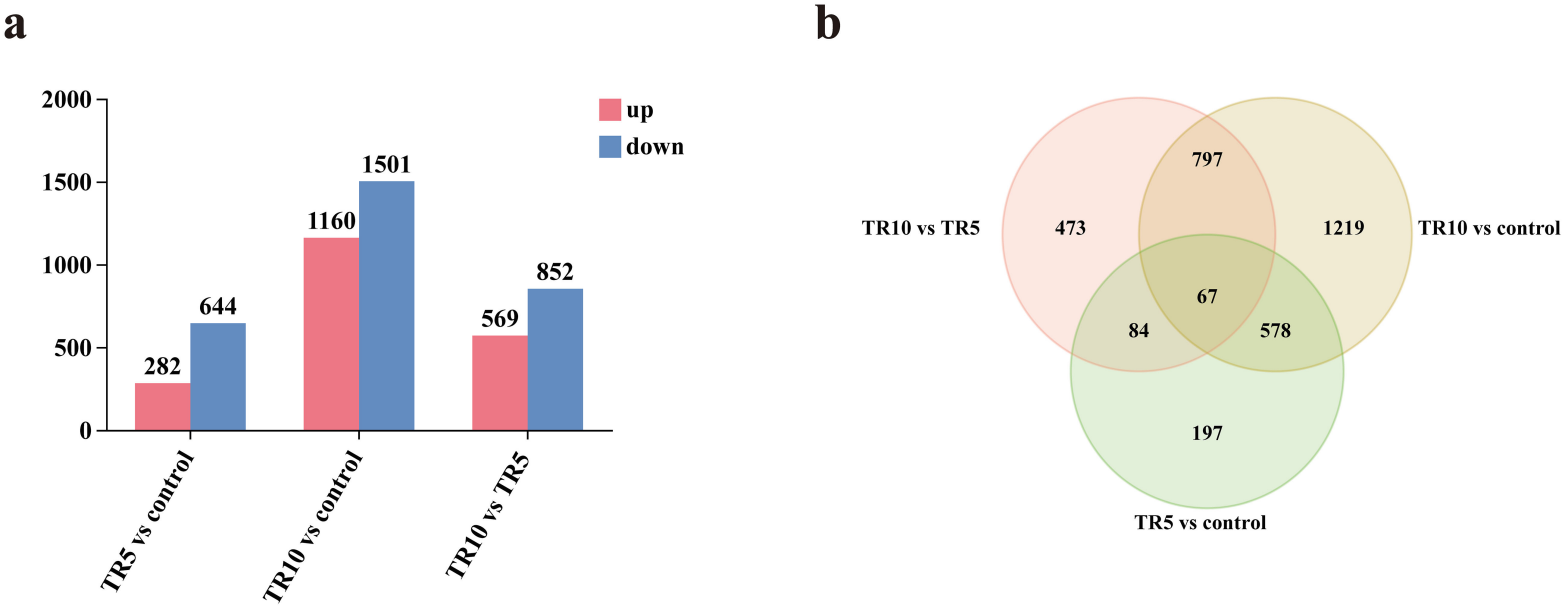
DEGs in grape callus after PEG treatment for different durations. **(a)** Upregulated and downregulated of the DEGs number. **(b)** Venn diagram analysis of DEGs across gene sets.

Differential gene expression analysis was performed using DESeq2 based on normalized expression data. Genes with |log_2_ fold change| ≥ 1 and adjusted *P*-value < 0.05 were defined as significantly DEGs. The results revealed that a large number of genes exhibited significant expression changes in grape callus in response to drought stress (**Figure 3A**). A total of 1,421, 2,661, and 926 DEGs were identified in the comparisons of TR10 vs TR5, TR10 vs control, and TR5 vs control, respectively. In the TR10 vs TR5 group, 569 genes were upregulated and 852 genes were downregulated. In the TR10 vs control group, 1,160 genes were upregulated and 1,501 genes were downregulated. In the TR5 vs control group, 282 genes were upregulated, whereas 644 genes were downregulated. Overall, the number of DEGs increased with the duration of drought treatment, indicating a progressively enhanced transcriptional response to stress. Venn diagram analysis further revealed that 67 DEGs were shared among different comparison groups (**Figure 3B**), suggesting the presence of a core set of genes consistently involved in drought response. Notably, several stress-responsive transcription factors, including members of the DREB, ERF109, and TGA families, were identified among these shared DEGs, indicating their potential involvement in grape drought adaptation under PEG treatment.

### GO and KEGG enrichment analysis of differentially expressed genes

**Figure 4:**
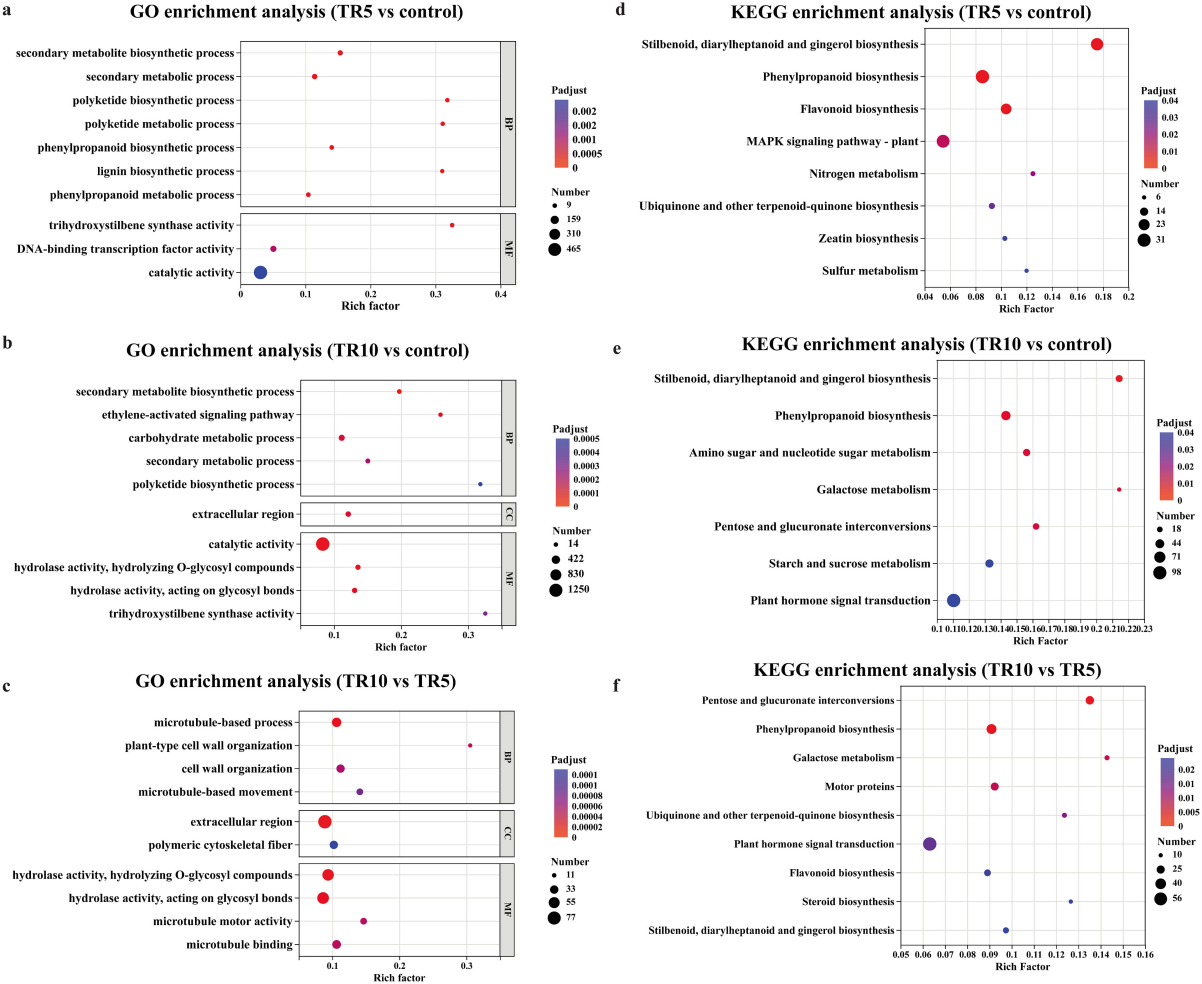
GO enrichment analysis and KEGG enrichment diagram of DEGs. **(a–c)** GO enrichment analysis. BP, biological process; CC, cellular process; MF, molecular function. The vertical axis represents the GO terms, and the horizontal axis represents the Rich factor. A larger Rich factor indicates a higher degree of enrichment. The size of each dot represents the number of genes/transcripts associated with the corresponding GO term, while the color of the dots corresponds to different ranges of adjusted *P*-values (Padjust). **(d–f)** KEGG pathway analysis. The vertical axis represents the pathway names, and the horizontal axis represents the Rich factor. A larger Rich factor indicates a higher degree of enrichment. The size of each dot represents the number of genes associated with the corresponding pathway, while the color of the dots corresponds to different ranges of adjusted *P*-values (Padjust).

To explore the functional characteristics of DEGs under drought stress, GO enrichment analysis was performed for the three comparison groups (TR10 vs TR5, TR10 vs control, and TR5 vs control) (**Figure 4A**). The DEGs were mainly classified into three major categories: biological process, cellular component, and molecular function.

In the TR10 vs TR5 and TR10 vs control groups, DEGs were significantly enriched in all three GO categories, whereas in the TR5 vs control group, enrichment was mainly observed in biological process. Across all comparisons, DEGs were predominantly associated with processes related to carbohydrate metabolism and secondary metabolite biosynthesis. In particular, secondary metabolite biosynthetic processes were consistently enriched, suggesting their important roles in drought stress response.

To further investigate the biological pathways involved, KEGG pathway enrichment analysis was conducted (**Figure 4B**). The results showed that DEGs were significantly enriched in pathways related to phenylpropanoid biosynthesis, suggesting that it plays a central role in mediating drought stress responses in grape callus. In addition, flavonoid biosynthesis and MAPK signaling pathways were enriched in the TR5 vs control group, while amino sugar and nucleotide sugar metabolism was mainly enriched in the TR10 vs control group.

### Identification of differentially expressed transcription factors under drought stress

**Figure 5:**
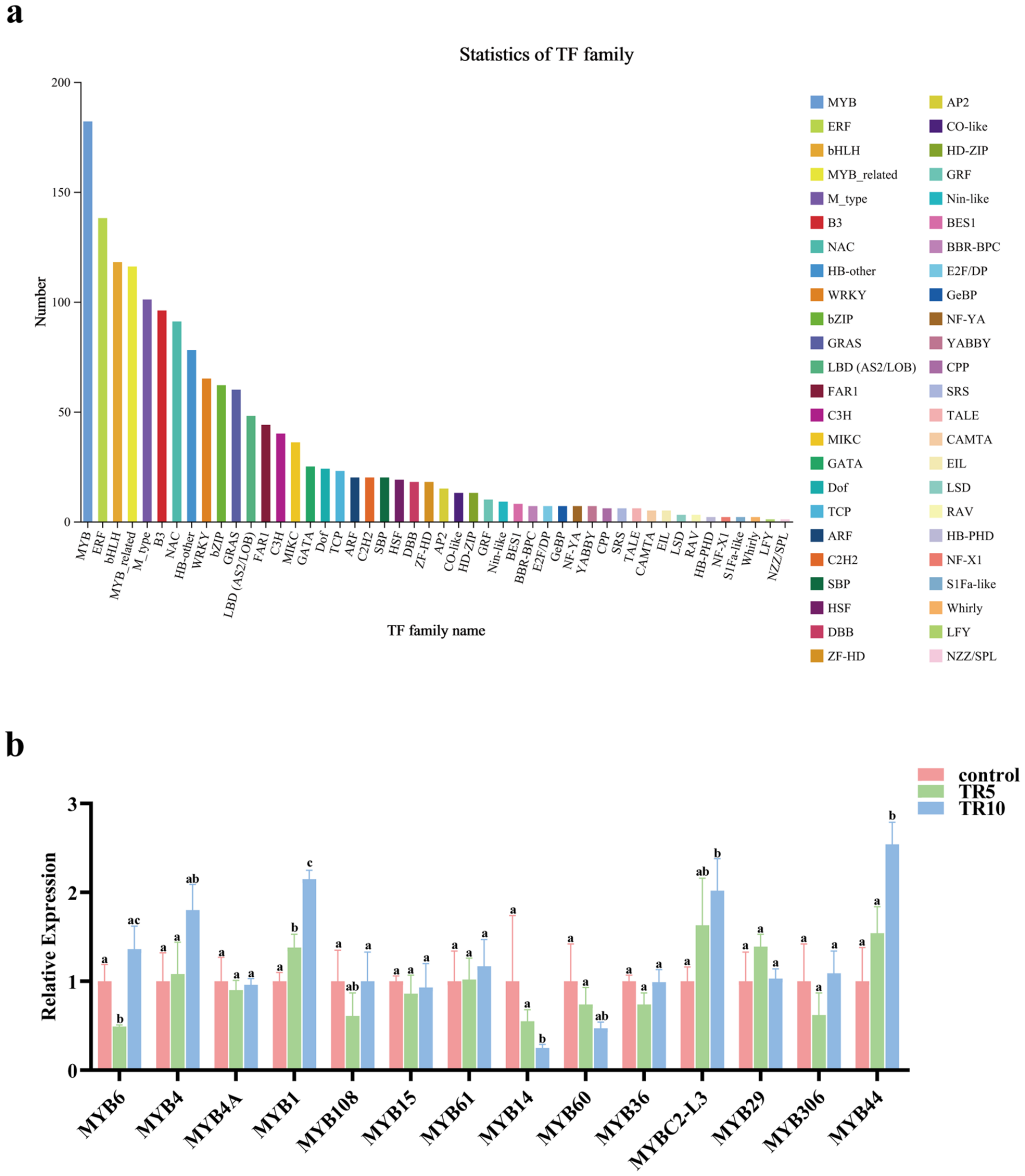
Identification of transcription factors. **(a)** Statistics of Transcription factor family. **(b)** *VvMYB* transcription factors expression. Data represent the mean ± SD of three biological replicates. Identical letters indicate no significant difference (*P* > 0.05).

To further investigate the regulatory mechanisms underlying drought stress responses, TFs were identified from the differentially expressed genes. A total of 48 TF families were detected in the transcriptome (**Figure 5A**), including well-known stress-responsive families such as MYB, WRKY, NAC, bZIP, and ERF. Among these, the MYB family represented the largest proportion of TFs (**Figure 5B**), suggesting its prominent role in regulating drought responses in grape. Members of the MYB family exhibited dynamic expression patterns across different stages of drought treatment, indicating their potential involvement in stress-responsive transcriptional regulation. Notably, *VvMYB1* showed significant differential expression under drought stress, particularly across multiple treatment stages. Therefore, *VvMYB1* was selected for further functional characterization.

### Identification of OE-*VvMYB1* transgenic callus

**Figure 6:**
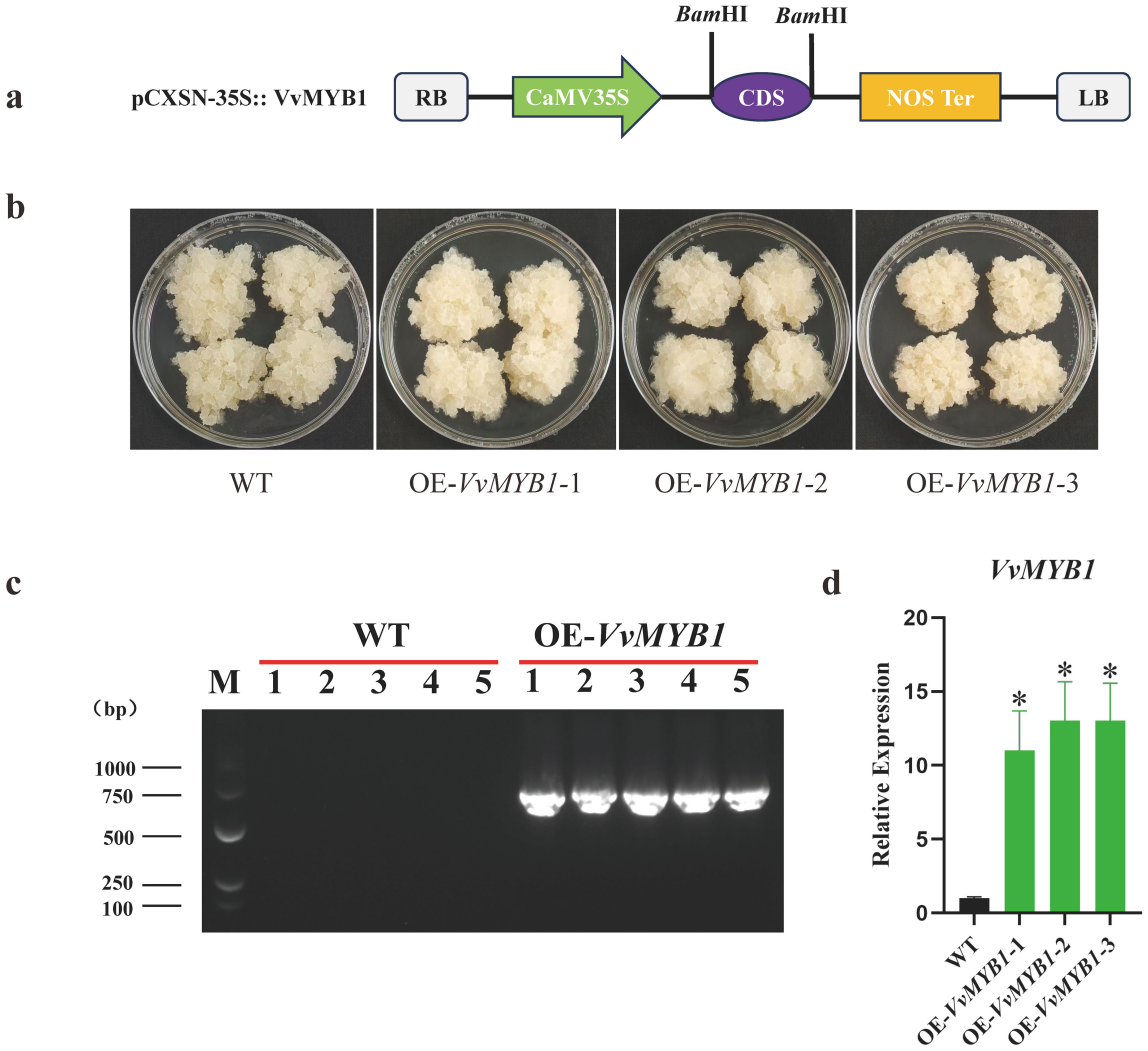
Identification of transgenic callus of OE-*VvMYB1.* **(a)** Schematic of the overexpression *VvMYB1* (OE-*VvMYB1*) vector. CaMV35S: promoter. NOS Ter: terminator. **(b)** The OE-*VvMYB1* transgenic grape calli. **(c)** The hygromycin detection of OE-*VvMYB1* transgenic grape calli. 2000 bp DNA Marker. **(d)** The expression level of *VvMYB1* in the OE-*VvMYB1* grape calli. Data is mean ± SD of three biological replicates. The independent sample *t*-est was used to compare the two groups of data. *P < 0.05.

Transgenic callus OE-*VvMYB1* was successfully generated via *Agrobacterium tumefaciens*-mediated transformation (**Figure 6**). The overexpression construct was based on the pCXSN vector, which contains a *Hyg* resistance gene (**Figure 6A**), allowing transgenic callus to be selected and maintained on Hyg-containing medium. PCR amplification of the *Hyg* gene was performed to verify the presence of the transgene. As shown in **Figure 6C**, a specific amplification band was detected in OE-*VvMYB1* callus, whereas no corresponding band was observed in WT callus, confirming successful transformation. Furthermore, qRT-PCR analysis revealed that the expression level of *VvMYB1* was significantly increased in OE-*VvMYB1* callus compared with WT (**Figure 6D**). Taken together, these results demonstrate that OE-*VvMYB1* transgenic callus was successfully obtained.

### Overexpression of *VvMYB1* enhances drought tolerance in grape callus

**Figure 7:**
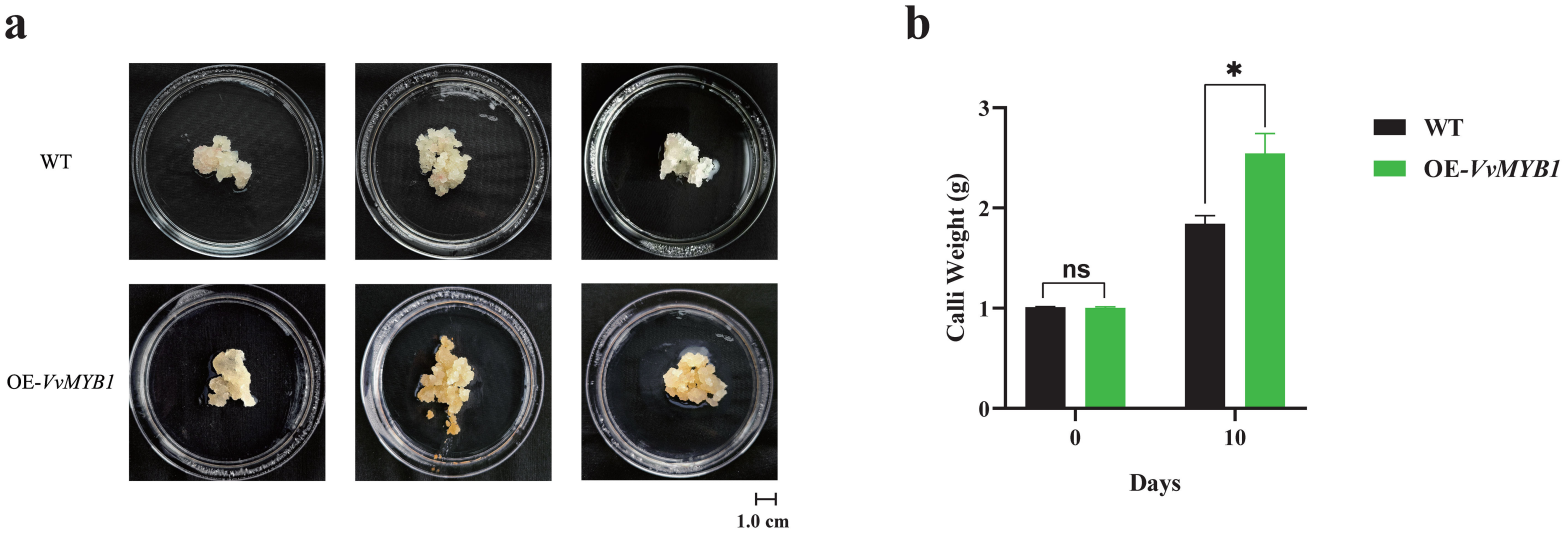
Comparison of morphological characteristics and weight of WT and OE-VvMYB1 callus tissues. **(a)** Morphological characteristics of the grape callus of WT and OE-*VvMYB1*. **(b)** Comparison of the weight of the grape callus between WT and OE-*VvMYB1*. Note: WT is callus of wild type grape. OE-*VvMYB1* is the strain overexpressing *VvMYB1*. Data is mean ± SD of three biological replicates. The independent sample t test was used to compare the two groups of data. ns: no significant difference; *P < 0.05.

Phenotypic differences between WT and OE-*VvMYB1* callus were evaluated under drought stress conditions (**Figure 7**). As shown in **Figures 7A**, OE-*VvMYB1* callus exhibited a larger size and better growth status compared with WT after drought treatment. Consistently, biomass analysis revealed that the fresh weight of OE-*VvMYB1* callus was significantly higher than that of WT (**Figure 7B**), indicating enhanced growth performance under stress conditions. These results suggest that overexpression of *VvMYB1* improves drought tolerance in grape.

### Overexpression of *VvMYB1* improves physiological responses under drought stress

**Figure 8:**
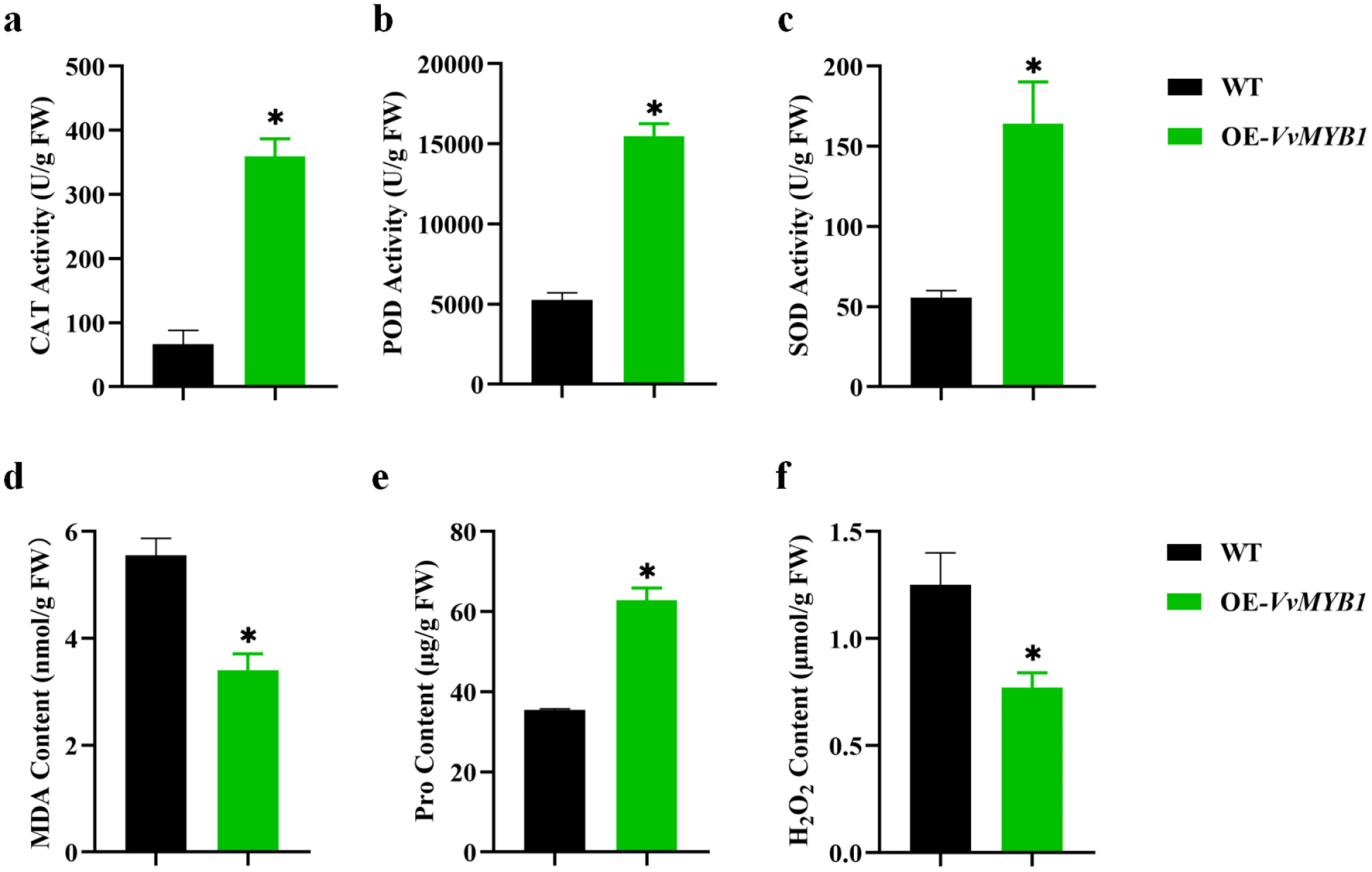
Comparison of enzyme activity and biochemical products in grape callus. The **(a–f)** show the changes in various physiological indicators of WT and OE-*VvMYB1* callus under drought treatment. CAT, catalase; POD, peroxidase; MDA, malondialdehyde; SOD, superoxide dismutase; Pro, proline; H_2_O_2_, hydrogen peroxide. Data is mean ± SD of three biological replicates. The independent sample t test was used to compare the two groups of data. *P < 0.05.

To further evaluate the physiological responses to drought stress, several stress-related parameters were measured in WT and OE-*VvMYB1* callus (**Figure 8**). The activities of antioxidant enzymes, including CAT, POD, and SOD, were significantly higher in OE-*VvMYB1* callus than in WT. In addition, the Pro content was markedly increased in OE-*VvMYB1* callus. In contrast, the levels of MDA and H_2_O_2_, which are indicators of oxidative damage, were significantly lower in OE-*VvMYB1* callus compared with WT. These results indicate that overexpression of *VvMYB1* enhances antioxidant capacity and reduces oxidative damage, thereby improving drought tolerance in grape.

### Overexpression of *VvMYB1* upregulates stress-responsive genes

**Figure 9:**
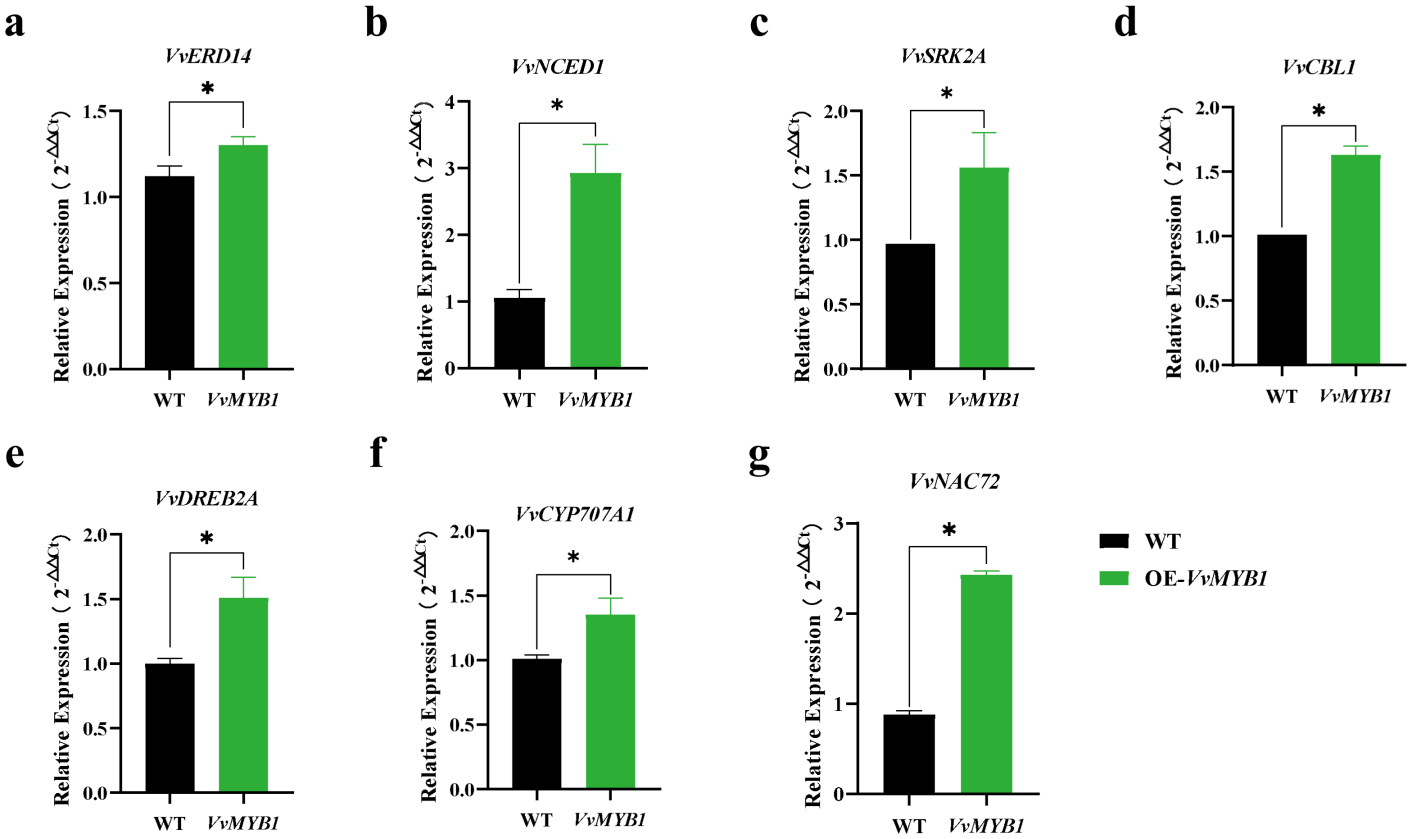
Relative expression of drought resistance genes. The **(a–g)** show the changes in the expression levels of stress-resistant related genes in WT and OE-*VvMYB1* under drought conditions. Data is mean ± SD of three biological replicates. The independent sample *t* test was used to compare the two groups of data. *P < 0.05.

To further investigate the molecular basis of enhanced drought tolerance, the expression levels of stress-responsive genes were analyzed by qRT-PCR (**Figure 9**). The results showed that the expression levels of *VvERD14*, *VvDREB2A*, *VvNAC72*, *VvSRK2A*, and *VvCBL1* were significantly higher in OE-*VvMYB1* callus compared with WT under drought stress conditions. These findings indicate that overexpression of *VvMYB1* promotes the transcription of multiple stress-related genes, suggesting its involvement in the regulation of drought-responsive gene networks.

## Discussion

Plants have evolved complex regulatory networks to cope with diverse environmental stresses, including drought, salinity, temperature extremes, and oxidative stress (Yang et al., 2022). As a perennial fruit crop with high economic value, grape (*Vitis vinifera*) is particularly sensitive to environmental fluctuations, and its growth and productivity are severely affected by abiotic stresses (Zhu et al., 2023). These stress responses are orchestrated by intricate transcriptional regulatory networks, in which multiple TF families, such as bZIP, WRKY, NAC, and MYB, play central roles in modulating downstream gene expression and physiological adaptation (Jacques et al., 2021).

Previous studies have demonstrated that diverse TFs participate in grape stress responses through distinct regulatory mechanisms. For instance, VvFHY3 promotes anthocyanin biosynthesis under high temperature by repressing VvbZIP17, while VvWRKY70 regulates isoprenoid and flavonoid biosynthesis by relieving transcriptional repression under heat stress (Wei et al., 2023; Sun et al., 2024). In addition, heterologous expression of VvWRKY28 enhances tolerance to cold and salt stress in *Arabidopsis* (Liu et al., 2022b). Several signaling-related genes have also been implicated in stress adaptation; for example, VaIAA3 modulates cold tolerance via auxin, ABA, and ethylene signaling pathways, whereas VaMAPKKK15 negatively regulates cold tolerance (Lu et al., 2024b, Feng et al., 2025). Moreover, VvSnRK2.7 contributes to drought tolerance by enhancing antioxidant enzyme activity and maintaining metabolic stability (Lan et al., 2024). Other TFs, such as VvWRKY18 and VvNAC17, have been reported to negatively or positively regulate drought tolerance, respectively (Zhang et al., 2022; Jin et al., 2025). Together, these findings highlight the complexity and diversity of transcriptional regulation in grape stress responses.

In the present study, Venn diagram analysis revealed 67 commonly differentially expressed genes across multiple drought treatment comparisons, implying the presence of a conserved transcriptional regulatory network associated with drought adaptation in grape callus. Among these shared DEGs, several well-characterized stress-responsive transcription factor families, including DREB, ERF109, and TGA, were consistently induced under PEG treatment. DREB and ERF family members are widely recognized as central regulators of abiotic stress signaling and downstream transcriptional reprogramming, whereas TGA transcription factors have been implicated in redox homeostasis, hormone signaling, and stress-associated defense responses (Lu et al., 2024a, Qiu et al., 2026; Zhang et al., 2026). The coordinated differential expression of these transcription factors suggests that grape activates a complex and integrated regulatory framework to cope with drought-induced cellular stress. Moreover, the enrichment of multiple stress-related TFs among the shared DEGs further highlights the robustness and biological relevance of the transcriptomic dataset generated in this study.

Among these TF families, MYB transcription factors represent one of the largest and most functionally diverse groups in plants and are widely involved in regulating abiotic stress responses (Zhang et al., 2025). Numerous studies across species have demonstrated that MYB TFs participate in drought tolerance by modulating hormone signaling, reactive oxygen species (ROS) homeostasis, and osmotic adjustment. For example, GmMYB14 regulates drought tolerance in soybean by modulating brassinosteroid levels, while GhMYB36 enhances drought resistance by promoting defense-related gene expression (Chen et al., 2021; Liu et al., 2022a). In addition, MYB TFs such as MaMYBR30, OsMYB2, and MdMYB108L have been shown to regulate stress tolerance through diverse pathways, including osmotic regulation, ion homeostasis, and transcriptional activation of stress-related genes (Du et al., 2022; Liu et al., 2024; Nie et al., 2025). Conversely, some MYB TFs function as negative regulators, such as FtMYB22, which suppresses stress tolerance via an ABA-dependent pathway (Zhao et al., 2022). These studies collectively suggest that MYB act as key regulators in coordinating plant stress responses.

In grape, MYBs have also been implicated in abiotic stress adaptation. For instance, overexpression of VhMYB2 enhances tolerance to drought and salinity (Ren et al., 2023b), and VhMYB60 improves tolerance to salt and cold stress (Chen et al., 2025). Additionally, VvMYB44–1 negatively regulates anthocyanin biosynthesis under high temperature by interfering with MBW complex formation (Leng et al., 2025). However, the functional role of VvMYB1 in drought stress response has remained largely unexplored.

In the present study, transcriptome analysis revealed that MYB were the most abundant TF family responding to drought stress, suggesting their prominent role in grape stress adaptation. Based on expression patterns, VvMYB1 was selected for further functional characterization. Our results demonstrated that overexpression of VvMYB1 significantly enhanced drought tolerance in grape callus, as evidenced by improved growth performance and increased biomass under stress conditions.

At the physiological level, OE-*VvMYB1* callus exhibited higher activities of antioxidant enzymes (SOD, POD, and CAT) and increased accumulation of proline, accompanied by reduced levels of MDA and H_2_O_2_. Similar physiological responses have also been reported in other drought-responsive grape MYB TFs, such as VhMYB2, suggesting that enhancement of antioxidant capacity may represent a common mechanism underlying MYB-mediated drought adaptation in grape (Ren et al., 2023a). These physiological changes were associated with improved oxidative stress tolerance and enhanced ROS-scavenging capacity in OE-*VvMYB1* callus. Since excessive ROS accumulation is a common consequence of drought-induced cellular damage, the reduced H_2_O_2_ and MDA levels observed in OE-*VvMYB1* lines suggest that VvMYB1 may participate in the maintenance of cellular redox balance during drought stress. However, the precise molecular mechanisms underlying VvMYB1-mediated regulation of antioxidant responses remain unclear and require further investigation. In addition, KEGG analysis revealed significant enrichment of phenylpropanoid and flavonoid biosynthesis pathways under drought stress. Previous studies have shown that MYB TFs frequently participate in the regulation of secondary metabolism, including flavonoid biosynthesis, which contributes to ROS scavenging and stress adaptation (Jiang et al., 2023; Hu et al., 2024; Bhatt et al., 2025). Therefore, it is possible that VvMYB1 may also be associated with these pathways during drought response. However, further studies are required to determine whether VvMYB1 directly regulates phenylpropanoid/flavonoid metabolism in grape.

Furthermore, the expression levels of several stress-responsive genes, including *VvERD14*, *VvDREB2A*, *VvNAC72*, *VvSRK2A*, and *VvCBL1*, were significantly upregulated in OE-*VvMYB1* callus. These genes are known to be involved in stress signaling and adaptive responses, suggesting that VvMYB1 may function as an upstream regulator in drought-responsive transcriptional networks. The coordinated upregulation of these genes, together with enhanced antioxidant activity, indicates that VvMYB1 contributes to drought tolerance through integrated regulation of stress-responsive pathways.

Taken together, our findings suggest that VvMYB1 acts as a positive regulator of drought stress response in grape, likely by modulating antioxidant defense systems and activating downstream stress-related genes. This study provides new insights into the molecular mechanisms underlying drought tolerance in grape and offers a potential candidate gene for improving stress resistance through genetic engineering and molecular breeding approaches. In the future, VvMYB1 may serve as a valuable genetic resource for the development of drought-tolerant grape cultivars in future grape breeding programs.

## Data Availability

The data presented in the study are deposited in NCBI with accession number PRJNA1471429.

## Supplementary material

Supplementary Table 1 The primers sequence.
