# Supplementary material for: Transcriptome analysis and functional validation reveal that VvMYB1 is a positive regulator of drought tolerance in grape
Source: Front Plant Sci. 2026 Jun 15;17:1853060. doi: 10.3389/fpls.2026.1853060 (PMC13310715; doi:10.3389/fpls.2026.1853060)
Supplement: Supplementary Table 1 — The primers sequence. [file Table1.docx]

Table S1. The primers sequence

| Primer | Sequence (5’-3’) |
| --- | --- |
| qPCR*-VvERD14-*F | TGATGAAGTAGTTCCACCACAG |
| qPCR*-VvERD14-*R | TTCTTCTCTTCTTCGGTCTTGG |
| qPCR*-VvDREB2A-*F | CGGGAGCCAAACAGAGGGAGT |
| qPCR*-VvDREB2A-*R | CATGGCCCTTGCAGCTTCGTC |
| qPCR*-VvNAC72-*F | GGGCTCGGGCAACTTCGACT |
| qPCR*-VvNAC72-*R | GGACGCCGGAGACCAACTCC |
| qPCR*-VvSRK2A-*F | GCCCCGGGAACTCACAGAAGC |
| qPCR*-VvSRK2A-*R | GCCTCCCCAGCCAAAGCCTC |
| qPCR*-VvCYP707A1*-F | TGCTGCTCAAGACACCACAG |
| qPCR*-VvCYP707A1*-R | CGGTTACCTTCCTCGTTAGAT |
| qPCR*-VvNCED1*-F | CAGCCGTGGCTCTCCTGTGG |
| qPCR*-VvNCED1*-R | TCGGTCTCCGGCTCTTCCCA |
| qPCR-*VvNAC72*-F | AAGAGGACGGGTAGGGTTAG |
| qPCR-*VvNAC72*-R | CAAATACTCGGCATAGGACA |
| qPCR-*VvACTIN*-F | ATTCTGGTGATGGTGTGAGT |
| qPCR-*VvACTIN*-R | GACAATTTCCCGTTCAGCAGT |
| qPCR-*VvNAC29*-F | AAAGCCACAGGGACAGACAA |
| qPCR-*VvNAC2*9-R | TCCTACATAGCACCCAATCA |
| qPCR-*VvSCL33*-F | GCTTGAGCCCAGATGGAGAT |
| qPCR-*VvSCL33*-R | GTCACTGCTACTGCCCGAAA |
| qPCR-*VvNAC21*-F | CACCAGGTTTCAGGTTCCAT |
| qPCR-*VvNAC21*-R | TTGCCCTATTTGTTCTAAGTCC |
| qPCR-*VvSCL21*-F | ATACTGCTCCATTCCTTTCC |
| qPCR-*VvSCL21*-R | TGATGGTGGCATTCACATAG |
| qPCR-*VvCSN5a*-F | CCTTTCTCGCAGTTGTTATT |
| qPCR-*VvCSN5a*-R | GGTCATCTGGAGGCTTGTAT |
| qPCR-*VvWRKY1*-F | GCAATGTGAAGAAGCAGGTC |
| qPCR-*VvWRKY1*-R | TGTTCAAAGTTCTCGGTGGG |
